# Notoginsenoside R1 for Organs Ischemia/Reperfusion Injury: A Preclinical Systematic Review

**DOI:** 10.3389/fphar.2019.01204

**Published:** 2019-10-17

**Authors:** Qiang Tong, Peng-chong Zhu, Zhuang Zhuang, Li-hui Deng, Zi-hao Wang, Hua Zeng, Guo-qing Zheng, Yan Wang

**Affiliations:** ^1^Department of Cardiology, The Second Affiliated Hospital and Yuying Children’s Hospital of Wenzhou Medical University, Wenzhou, China; ^2^Department of Neurology, The Second Affiliated Hospital and Yuying Children’s Hospital of Wenzhou Medical University, Wenzhou, China

**Keywords:** notoginsenoside R1, ischemia, reperfusion, organ, preclinical systematic review, meta-analysis

## Abstract

Notoginsenoside R1 (NGR1) exerts pharmacological actions for a variety of diseases such as myocardial infarction, ischemic stroke, acute renal injury, and intestinal injury. Here, we conducted a preclinical systematic review of NGR1 for ischemia reperfusion (I/R) injury. Eight databases were searched from their inception to February 23rd, 2019; Review Manager 5.3 was applied for data analysis. CAMARADES 10-item checklist and cell 10-item checklist were used to evaluate the methodological quality. Twenty-five studies with 304 animals and 124 cells were selected. Scores of the risk of bias in animal studies ranged from 3 to 8, and the cell studies ranged from 3 to 5. NGR1 had significant effects on decreasing myocardial infarct size in myocardial I/R injury, decreasing cerebral infarction volume and neurologic deficit score in cerebral I/R injury, decreasing serum creatinine in renal I/R injury, and decreasing Park/Chiu score in intestinal I/R injury compared with controls (all P < 0.05 or P < 0.01). The multiple organ protection of NGR1 after I/R injury is mainly through the mechanisms of antioxidant, anti-apoptosis, and anti-inflammatory, promoting angiogenesis and improving energy metabolism. The findings showed the organ protection effect of NGR1 after I/R injury, and NGR1 can potentially become a novel drug candidate for ischemic diseases. Further translation studies are needed.

## Introduction

Ischemia and reperfusion (I/R) injury, featuring as an interruption of organ blood flow and the following re-oxygenation after reperfusion, is a common pathological phenomenon in ischemic diseases. These mainly include myocardial infarction (MI), ischemic stroke, acute renal injury, and intestinal injury (Eltzschig and Eckle 2011). Although these pathological processes are involved in a variety of diseases, these diseases share common molecular mechanisms. The main mechanisms of I/R injury include inflammation (Chen and Nuñez, 2010), oxidative stress (Ohsawa et al., 2007), apoptosis (Shiva et al., 2007), energy metabolism disorder (Wang and Ma, 2018), microvascular dysfunction (Eltzschig and Collard, 2004), and leucocyte-endothelial cell adhesion (Eltzschig and Eckle 2011). Owing to the chronic organ injury, the ischemia organ will ultimately develop into the pathological outcomes with tissue fibrosis and organ failure (Friedman et al., 2013). In 2016, the two main ischemic diseases, ischemic heart disease (IHD) and stroke, were the leading causes of human death globally, accounting for more than 85.1% of all cardiovascular and cerebrovascular diseases and death (Murray Christopher 2017). Over the past three decades, great progress has been made in the therapy of ischemic diseases, especially in MI and ischemic stroke. Nevertheless, there exist disadvantages in safety and efficacy in main and promising therapy approaches. In acute management, the timely revascularization therapies such as percutaneous coronary intervention (PCI) and thrombolysis can recover the supply of oxygen and blood for ischemic organs or tissues. However, irreversible ischemia from delayed administration and reperfusion injury can result in chronic organ failure and a shortened lifespan (Alex et al., 2017). Currently antiplatelet drugs have good effects on anti-platelet adhesion and aggregation in clinic, but they have little efficacy in energy metabolism disorder and oxidative stress (Han et al., 2017). Studies have shown that ischemic preconditioning and ischemic postconditioning can reduce MI size caused by I/R injury, whereas the mechanism of the conditioning phenomenon with the most robust cardioprotective procedure through interventions reducing MI size is still largely unknown in the human heart (Heusch and Rassaf 2016; Heusch and Gersh, 2016; Heusch, 2017). Currently, recanalization therapy for acute ischemic stroke (AIS) is mainly through recombinant tissue plasminogen activator (rt-PA). However, rt-PA is accepted by a minority of patients due to narrow time window of thrombolysis and hemorrhage (Hacke et al., 2004; Khandelwal et al., 2016). Unfortunately, the reocclusion after thrombolysis led to neurological function impairment and higher in-hospital mortality (Lee et al., 2001). Considering the limitations of these therapies, we need to seek new therapy to improve organ damage induced by I/R injury.

**Figure 1:**
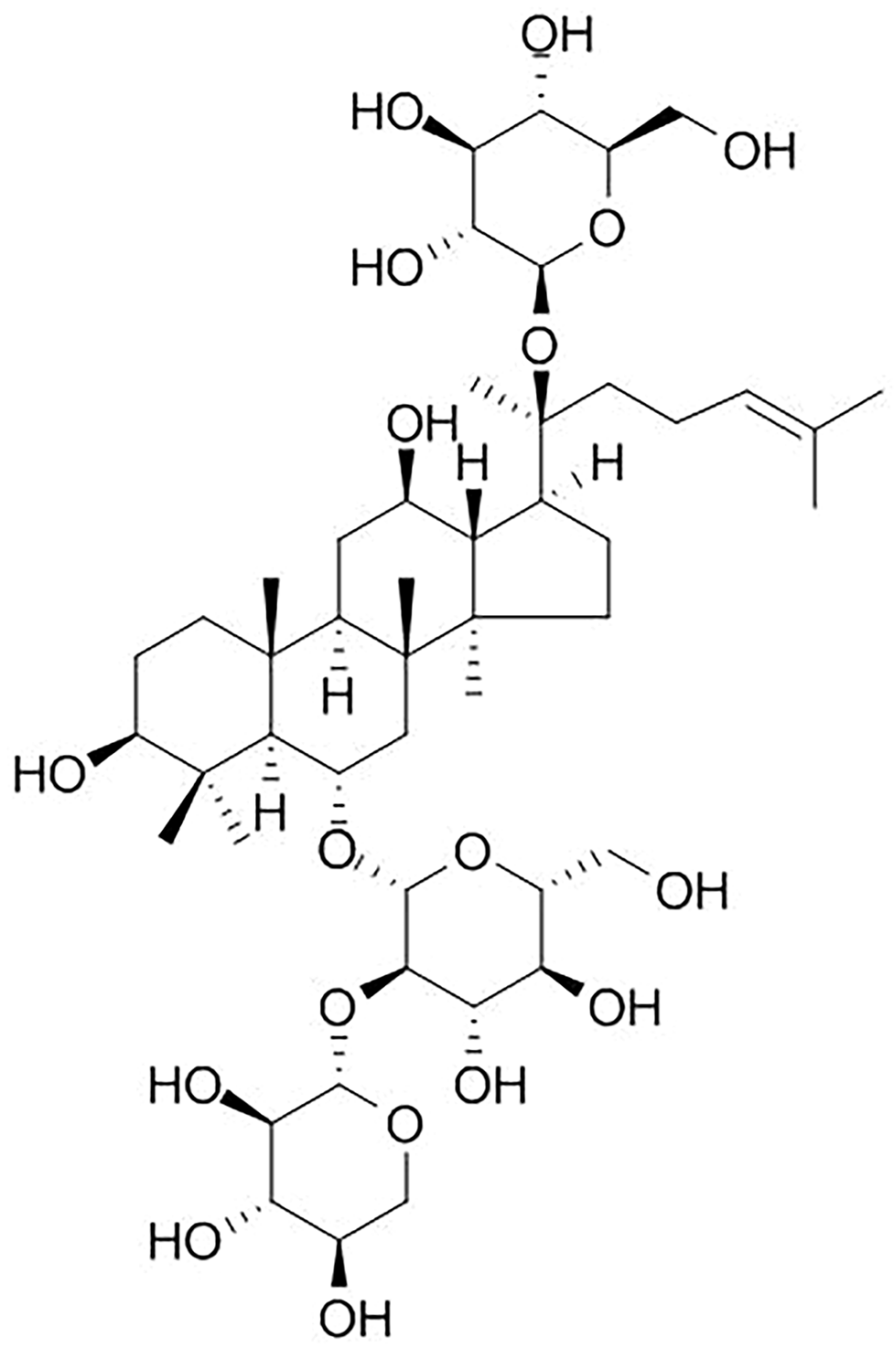
Chemical structure of notoginsenoside R1.

*Panax notoginseng* (*P. notoginseng*), one of the most valuable traditional Chinese medicine (TCM), is derived from the roots and rhizomes of *P. notoginseng* (Burkill) F.H. Chen (Ng, 2006; Peng et al., 2018). Over the past several centuries, *P. notoginseng* showed good efficacy in controlling internal and external bleeding and improving blood stasis (Wang T. et al., 2016). With the advancing of pharmacology, the studies of *P. notoginseng* demonstrate that it is widely used in cardiovascular diseases (CVDs) mainly because of its vasodilatory and antihypertensive functions (Yang et al., 2014). Notoginsenoside R1 (NGR1) (the specific chemical structure of NGR1 is shown in **Figure 1**) is the main effective component isolated from *P. notoginseng*, and NGR1 belongs to protopanaxatriol (PPT) type of saponins (Leung et al., 2007). Previous studies indicated that NGR1 is easily dissolved in water, but shows low bio-availability and poor permeability in the gastrointestinal tract (Liang and Hua, 2005; Liu et al., 2009; Ruan et al., 2010). In absorption property, Liang et al. (2005) revealed that the optimal site for absorption of NGR1 is the upper extremity of the intestine. Guo et al. (2014) first described the detection of NGR1 in brain tissue by the way of nasally applying drugs. To improve the oral bioavailability of NGR1, sodium N-[8-(2-hydroxybenzoyl) amino] caprylate (SNAC, a novel absorption enhancer) was used (Li et al., 2018). It has been reported that NGR1 significantly improves prognosis of other disease models such as atherosclerosis, diabetic nephropathy, and diabetic cardiomyopathy (Su et al., 2015; Zhang et al., 2018; Zhang B. et al., 2019). NGR1 also plays a protective role in ischemic diseases (Liu et al., 2010; Li et al., 2014; Yu et al., 2016; Tu et al., 2018). Although NGR1 has been widely used for the treatment of ischemic diseases, the efficacy and mechanisms of NGR1 for ischemic organs such as heart, cerebral, kidney, intestinal, and liver have not been systematically analyzed. Animal experiment, the most important approach of basic research, is a bridge between the bench and bedside (Olesen et al., 2012). The conclusions derived from preclinical studies are of little evaluation, and using these conclusions as inadequate evidence for conducting clinical trials has resulted in a high cost in clinical research or withdrawal of the drug from the market later (Perel et al., 2006). Systematic reviews (SRs) are usually used in clinical study, which provided available resources for performing clinical practice guidelines and policies. SR of preclinical studies also plays a significant role in many aspects, including: 1) improving the methodological quality of studies, 2) choosing suitable animal models, 3) translating the experimental data from preclinical to clinical, and 4) implementing the 3Rs (reduction, replacement, and refinement) (De Vries et al., 2014). In addition, SR of preclinical experiments is of significance to elucidate the mechanism and treatment of human diseases (Sena et al., 2014). SR can further evaluate the preclinical evidence objectively and reduce the bias of experimental results (Roberts et al., 2002; Perel et al., 2006). SRs for preclinical study that could offer crucial information for clinical research are quite scarce (Judith et al., 2014; Tsujimoto et al., 2017). Thus, we conducted an SR on preclinical studies of NGR1 for I/R injury.

## Methods

### Search Strategy

We searched PubMed, Cochrane Library, EMBASE, Web of Science, Chinese National Knowledge Infrastructure (CNKI), Wanfang Data Information Site, VIP information database, and Chinese Biomedical Literature Database from their inception to February 23rd, 2019. The following terms were used: 1) “notoginsenoside” AND “infarction OR ischem* OR reperfusion” limited on animals; and 2) “notoginsenoside” AND “Oxygen–glucose deprivation OR hypoxia” limited on cells.

### Inclusion Criteria/Exclusion Criteria

Inclusion criteria were prespecified as follows: 1) experimental animal models of I/R; 2) experimental cell models established by oxygen and glucose deprivation/reoxygenation (OGD/R); 3) treatment group received the NGR1 therapy merely; 4) control group received vehicle, non-functional liquid with equal volume, no treatment or positive control; 5) the primary outcome measures were MI size, creatine kinase (CK) or creatine kinase isoenzymes MB (CKMB), left ventricular ejection fraction (LVEF) and cardiac troponin I/T (cTnI/T) in myocardial I/R studies; cerebral infarct volume and neurologic deficit score in cerebral I/R studies; myeloperoxidase (MPO), glucose/water clearance and Park/Chiu score in intestinal I/R studies; MPO, serum creatinine (Scr), glomerular filtration rate (GFR), and blood urea nitrogen (BUN) in renal I/R studies, and alanine aminotransferase (ALT) and aspartate aminotransferase (AST) in liver I/R studies; cell viability, lactate dehydrogenase (LDH), superoxide dismutase (SOD), malondialdehyde (MDA), apoptosis rate and/or TUNEL positive rate in cell models; 6) the secondary outcome measures were mechanisms of NGR1 intervention in both animal and cell models. Exclusion criteria were prespecified as follows: 1) treatment group received non-NGR1; 2) comparing NGR1 with other herbal medicine or herbal active compounds; 3) no control group; 4) master dissertation or doctoral dissertation; 5) case report or review; 6) NGR1 for other disease models; 7) NGR1 in combination with other drugs; 8) duplicate publication.

### Data Extraction

A data extraction form was used to collect the following items from each included study: 1) first author, year of publication; 2) detailed information about the experimental subjects such as animal species, number, sex and weight, and cell number, organism, age, tissue, and primary/subcultured; 3) administration method and duration of NGR1 treatment; 4) the types and administration methods of anesthetics; 5) the outcome measures including type, timing, and mean and standard deviations.

Only the last time point and the highest dose were recorded if there were many different time points of outcome measures or the experimental animals received different doses of the drug. The data were measured by the digital ruler software if the data were presented with graphs. Further information was retrieved by contacting with the authors when the primary data were incomplete.

### Assessment of the Risk of Bias

Minor modified CAMARADES 10-item scale was used to assess the risk of bias in animal studies (Macleod et al., 2004). The modified item is the use of anesthetic with no intrinsic organ-protective activity. Our newly developed scale specially designed for cell studies was used for the assessment of risk of bias in cell experiments (Bao et al., 2018).

### Statistical Analysis

All data analysis was implemented by RevMan 5.3 (https://community.cochrane.org). We calculated the standard mean difference (SMD) with 95% confidence intervals (CIs). Heterogeneity was assessed using the Cochrane Q-statistic test (P < 0.05 was considered statistically effective) and the I^2^-statistic test. A random-effects model would be adopted if I^2^ > 50%, which indicates substantial heterogeneity. Conversely, a fixed-effects model would be used if I^2^ < 50%. Sources of heterogeneity were searched as far as possible, and subgroup analysis was carried out when necessary. The sensitivity analysis was performed in order to improve the robustness of the results.

## Results

### Study Selection

**Figure 2:**
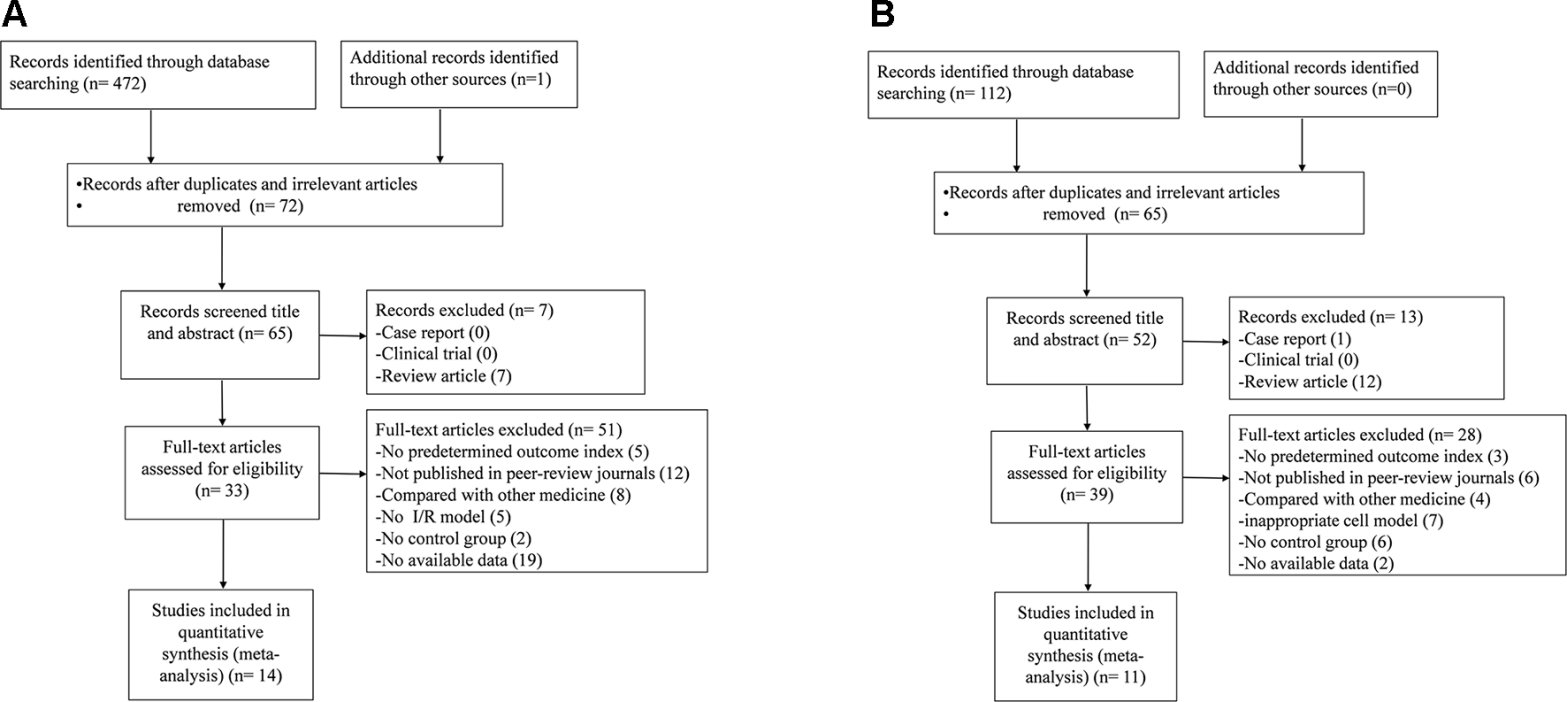
Summary of the process for identifying candidate studies. **(A)** Search strategy for animal experiments: 473 potentially relevant studies were identified; after removal of duplicates and the application of inclusion and exclusion criteria, 14 studies were included in the meta-analysis. **(B)** Search strategy for cell experiments: 112 potentially relevant studies were identified; after removal of duplicates and the application of inclusion and exclusion criteria, 11 studies were included in the meta-analysis.

For animal studies, a total of 473 potentially relevant hints were identified, of which 401 were duplicated. After screening of the titles and abstracts, seven studies were excluded because of the following reasons: 1) case report; 2) clinical trial; and 3) review article. Then secondary screening was conducted by reading the full text of the remaining 65 studies; 51 studies were excluded because of at least one of the following reasons: 1) failed to obtain full text; 2) non-NGR1 treatment; 3) inappropriate animal model; 4) compared with Chinese herbal medicine or herbal active compounds; 5) no control group; 6) master dissertation or doctoral dissertation; and 7) no available data. Finally, 14 papers (Liu et al., 2010; Deng and Lai, 2013; Han, 2014; He et al., 2014; Li et al., 2014; Meng et al., 2014; Yu et al., 2014; Dong et al., 2015; Xia et al., 2015; Wang et al., 2016; Yu et al., 2016; Zhao et al., 2017; Zou et al., 2017; Tu et al., 2018) were included (**Figure 2A**).

For cell studies, a total of 112 potentially relevant hints were identified, of which 47 were duplicated. After screening of the titles and abstracts, 13 studies were excluded because of the following reasons: 1) case report; 2) clinical trial; and 3) review article. Then secondary screening was conducted by reading the full text of the remaining 52 studies; 28 studies were excluded because of at least one of the following reasons: 1) failed to obtain full text; 2) non-NGR1 treatment; 3) inappropriate cell model; 4) compared with Chinese herbal medicine or herbal active compounds; 5) no control group; 6) master dissertation or doctoral dissertation; and 7) no available data. Finally, 11 papers (He et al., 2014; Meng et al., 2014; Wan et al., 2015; Yu et al., 2016; Zhou et al., 2016; Wang et al., 2016; Hou et al., 2017; Wang et al., 2017; Zhou et al., 2017; Liu et al., 2019; Tu et al., 2018) were included (**Figure 2B**).

### Characteristics of Included Studies

#### Animal Experiments

Fourteen animal experiments between 2010 and 2018 were included. Five studies (Deng and Lai, 2013; Han, 2014; Yu et al., 2014; Dong et al., 2015; Zhao et al., 2017) were published in Chinese, and nine studies (Liu et al., 2010; He et al., 2014; Li et al., 2014; Meng et al., 2014; Xia et al., 2015; Wang et al., 2016; Yu et al., 2016; Zou et al., 2017; Tu et al., 2018) were published in English. Eleven studies (Liu et al., 2010; Deng and Lai, 2013; Han, 2014; He et al., 2014; Li et al., 2014; Meng et al., 2014; Dong et al., 2015; Xia et al., 2015; Yu et al., 2016; Zhao et al., 2017; Zou et al., 2017) used healthy adult Sprague-Dawley (SD) male rats, while one study (Yu et al., 2014) used male Wistar rat and two studies (Wang et al., 2016; Tu et al., 2018) used 7-day-old SD rats. The body weight of adult SD rats ranged from 180 to 300 g. To induce anesthesia, pentobarbital were used in five studies (Liu et al., 2010; He et al., 2014; Yu et al., 2014; Xia et al., 2015; Zhao et al., 2017); chloral hydratein in two studies (Dong et al., 2015; Zhou et al., 2017); isofluranein in two studies (Wang et al., 2016; Tu et al., 2018); urethane in one study (Yu et al., 2016); ulatanin in one study (Han, 2014); ketaminein in one study (Meng et al., 2014); and noanesthetic in one study (Deng and Lai, 2013). To establish animal models of myocardial I/R injury, ligation of left anterior descending coronary artery (LAD) was used in four studies (Han, 2014; He et al., 2014; Yu et al., 2014; Xia et al., 2015); ligation of LAD for 40 min followed by 60 min of reperfusion in one study (Yu et al., 2016); ligation of LAD for 30 min followed by 30, 60, and 90 min of reperfusion in one study (Han, 2014). One study (Deng and Lai, 2013) reported that AMI model induced by injecting pituitrin (0.65 U/kg) into sublingual. To establish cerebral I/R injury models, occlusion of middle cerebral artery (MCA) was used in three studies (Meng et al., 2014; Dong et al., 2015; Zhao et al., 2017); ligation of common carotid artery (CCL) in two studies (Wang et al., 2016; Tu et al., 2018); and bilateral common carotid artery occlusion (BCCAO) in one study (Zou et al., 2017). One study (Liu et al., 2010) induced renal I/R animal models by clamping left renal artery and vein, and one study (Li et al., 2014) induced intestinal I/R animal models by clamping superior mesenteric artery. For outcome measures, the MI size was used in four studies (Deng and Lai, 2013; Han 2014; He et al., 2014; Xia et al., 2015), serum CK in three studies (Deng and Lai, 2013; Xia et al., 2015; Yu et al., 2016), serum MDA in two studies (Xia et al., 2015; Yu et al., 2016), cerebral infarction volume in five studies (Meng et al., 2014; Dong et al., 2015; Wang et al., 2016; Zou et al., 2017; Tu et al., 2018), neurologic deficit score in two studies (Meng et al., 2014; Dong et al., 2015), serum creatinine in one study (Liu et al., 2010), and Park/Chiu score in one study (Li et al., 2014). The detailed characteristics of the included studies were generalized in **Table 1**.

**Table 1:** Characteristics of the 14 included animal studies.

Study (years)	Species (sex; n = experimental/control group)	Weight	Model(method)	Anesthetic	Treatment group(Method to astraddle sides)	Control group	Outcome index (time)	Intergroup differences
Deng and Lai 2013	SD rats (Half male and female; 10/10)	180–220 g	Sublingual vein injection of pituitrin	—	NGR1 (10 mg/kg/day; i.g.) for 4 days before ischemia and 7 days after ischemia	Negative control group, isometric normal saline (i.g.) for 4 days before ischemia and 7 days after ischemiaPositive control group, diltiazem (i.g.) for 4 days before ischemia and 7 days after ischemia	1. ST-segment and inversion rate of T-wave 2. AST 3. CK 4. CK-MB 5. LDH 6. LDH1 7. Myocardial infarct size 8. Bcl-2 9. Bax	1. P < 0.01 2. P < 0.05 3. P < 0.05 4. P < 0.05 5. P < 0.05 6. P < 0.05 7. P < 0.05 8. P < 0.01 9. P < 0.01
Han, 2014	SD rats (male; 6/6)	240–260 g	Block LAD 30 min after reperfusion	20%Ulatan (1.25 g/kg)	NGR1 (5 mg/kg/h; i.v.) 20 min before ischemia	Intravenous infusion of the equal volume of normal saline	1. Venular RBC velocity (%) 2. Albumin leakage (%) 3. Coronary blood flow (%) 4. Myocardial infarct size 5. Heart rat (bpm) 6. LVDP 7. LVSP 8. +dP/dtmax 9. − dP/dtmax 10. MPO 11. ICAM-1 12. CD18 13. Positive percent of TUNEL (%) 14. ATP 15. ADP 16. AMP 17. ADP/ATP 18. AMP/ATP 19. ATPα/GAPDH 20. ATP5D/GAPDH 21. ATPβ/GAPDH 22. ZO-1/GAPDH 23. VE/GAPDH 24. JAM-1/GAPDH 25. Claudin-5/GAPDH 26. Cav-1/GAPDH 27. Cav-3/GAPDH 28.p-Src/Src 29.Src/GAPDH	1. P < 0.05 2. P < 0.05 3. P < 0.05 4. P < 0.05 5. P > 0.05 6. P > 0.05 7. P < 0.05 8. P < 0.05 9. P < 0.05 10. P < 0.05 11. P < 0.05 12. P < 0.05 13. P < 0.05 14. P < 0.05 15. P > 0.05 16. P > 0.05 17. P < 0.05 18. P < 0.05 19. P > 0.05 20. P < 0.05 21. P > 0.05 22. P < 0.05 23. P < 0.05 24. P < 0.05 25. P < 0.05 26. P < 0.05 27. P < 0.05 28. P < 0.05 29. P > 0.05
Yu et al., 2014	Wistar rats (male; 13/13)	220–280 g	Block LAD	1% Pentobarbital sodium(40 mg/kg)	NGR1 (2.5 mg/kg/day; i.p.)for 4 weeks after ischemia	Intraperitoneal injection of equal volume of saline after ischemia	1. MVC 2. MVD 3. VEGF 4. bFGF	1. P < 0.05 2. P < 0.05 3. P < 0.05 4. P < 0.05
He et al., 2014	SD rats (male; 8/8)	240–260 g	Block LAD 30 min after reperfusion	2% Pentobarbital sodium	NGR1 (5mg/kg/h; i.v.) for 30 min before ischemia; 30 min during ischemia; and 90 min after ischemia	Continuous injection of saline (1 ml/h)	1. AAR/LV 2. Myocardial infarct size/AAR 3.+dP/dtmax 4.− dP/dtmax 5. LVSP 6. LVDP 7. TUNEL-positive 8. Bcl-2/Bax 9. Cleaved caspase-3/procaspase-3 10. ATP 11. AMP 12. P-AMPK/β-actin 13. ATP synthase-α/β-actin 14. ATP synthase-β/β-actin 15. ATP 5D/β-actin 16. ROCK/β-actin 17. P-MYPT1/MYPT1	1. P > 0.05 2. P < 0.05 3. P > 0.05 4. P < 0.05 5. P > 0.05 6. P > 0.05 7. P < 0.05 8. P < 0.05 9. P < 0.05 10. P < 0.05 11. P > 0.05 12. P < 0.05 13. P > 0.05 14. P > 0.05 15. P < 0.05 16. P < 0.05 17. P < 0.05
Xia et al., 2015	SD rats (male; 6/6)	250–300 g	Block LAD 30 min after reperfusion	Pentobarbital sodium (30 mg/kg)	NGR1 (60 mg/kg; i.g.) for 5 days	No treatment	1. Myocardial infarct size 2. CK 3. LDH 4. T-SOD 5. MDA 6. IL-1β 7. IL-8 8. TNF-α 9. p-NF-κBP65/NF-κBP65 10. p-IκBα/IκBα 11. VDUP1/GAPDH	1. P < 0.01 2. P < 0.01 3. P < 0.05 4. P < 0.001 5. P < 0.01 6. P < 0.01 7. P < 0.01 8. P < 0.01 9. P < 0.01 10. P < 0.01 11. P < 0.01
Yu et al., 2016	SD rats (male; 10/10)	200–220 g	The isolated Langendorﬀ-perfused rat hearts received ischemia/reperfusion(40 min/60 min)	Urethane	NGR1 (20 μM) for 15 min before the ischemia	No treatment	1. LVSP 2. Heart rate 3. +dp/dtmax 4. -dp/dt min 5. MDA 6. SOD 7. CAT 8. CK 9. GSH-Px activities 10. P-JNK/JNK 11. CHOP/β-actin 12. Bcl-2/BAX 13. GRP78/β-actin 14. P-PERK/PERK 15. P-eIf2α/eIf2α 16. IRE1/β-actin 17. ATF6/β-actin 18. Caspase-12/β-actin 19. BAX/β-actin	1. P < 0.01 2. P < 0.05 3. P < 0.01 4. P < 0.01 5. P < 0.01 6. P < 0.01 7. P < 0.01 8. P < 0.001 9. P < 0.001 10. P < 0.001 11. P < 0.001 12. P < 0.001 13. P < 0.01 14. P < 0.001 15. P < 0.001 16. P < 0.001 17. P < 0.01 18. P < 0.001 19. P < 0.001
Meng et al., 2014	SD rats (male; 40/40)	250–300 g	MCAO	Ketamine (80 mg/kg; i.p.)	NGR1 (20 mg/kg; i.p) before ischemia	Given the same amount of saline	1. Infarction volumes 2. Neurologic deficit score 3. TUNEL-positive cells rate 4. Caspase-3 activity 5. NADPH oxidase activity 6. Superoxide levels 7. Mitochondrial superoxide levels 8. MDA 9. Protein carbonyl levels 10. 8-OHdG levels 11. HO-1 activity	1. P < 0.01 2. P < 0.01 3. P < 0.01 4. P < 0.01 5. P < 0.01 6. P < 0.01 7. P < 0.01 8. P < 0.01 9. P < 0.01 10. P < 0.01 11 . P< 0.01
Dong et al., 2015	SD rats (male; 8/8)	180–200 g	MCAO	10% Chloral hydrate (300 mg/kg; i.p.)	NGR1 (7.0 mg/kg; i.p.) for 14 days after ischemia	Given the same amount of saline	1. Infarction volumes 2. Neurologic deficit score 3. Population spike 4. Escape latency 5. Target quadrant dwell time	1. P < 0.01 2. P < 0.01 3. P < 0.01 4. P < 0.01 5. P < 0.01
Wang et al., 2016	7-day-old SD rats (male; 5/5)	–	The common carotid artery (CCL)	Isoflurane (2.5%)	NGR1 (15 mg/kg· 12 h; i.p.) after CCL; before exposure to the hypoxic environment	No treatment	1. Infarction volumes 2. Ratio of GRP78/β-actin 3. Ratio of P-PERK/PERK 4. Ratio of P-IRE1α/IRE1α 5. Ratio of CHOP/β-actin	1. P < 0.05 2. P < 0.05 3. P < 0.05 4. P < 0.05 5. P < 0.05
Zhao et al., 2017	SD rats (male; 10/10)	–	MCAO	3% Pelltobarbitalum Natricum (0.2 ml/100 g)	NGR1 (5 mg/ml; i.v.) for 3 days	Given the same amount of saline	1. The number of TUNEL-positive cells 2. TNF-α mRNA	1. P < 0.05 2. P < 0.05
Zou et al., 2017	SD rats(male; 15/15)	250–300 g	BCCAO (ischemia; 20 min; reperfusion; 3 h)	Chloral hydrate (350 mg/kg; i.p.)	NGR1 (100 mg/kg; i.g.) after ischemia	Intragastric administrationof 0.5 ml saline	1. Cerebral infarction size 2. Relative expression of BDNF mRNA 3. Relative expression of Bcl-2 to β-actin 4. Relative expression of Bax to β-actin	1. P < 0.01 2. P < 0.01 3. P < 0.01 4. P < 0.01
Tu et al., 2018	7-day-old SD rats (male; 9/9)	–	CCL	Isoflurane (2.5%)	NGR1 (15 mg/kg; i.p.; q12 h) for 2 days after ischemia	Not mentioned	1. The water content of brain tissue 2. Volume of brain infarction 3. TUNEL positive nuclei 4. Brain weight ratio 5. The score of balance beam 6. Percent in the target quadrant 7. PI3K/β-actin 8. P-Akt/T-Akt 9. P-mTOR/T-mTOR 10. P-P70S6K/P70S6K 11. P-4EBP-1/4EBP-1 12. P-JNK/T-JNK 13. P-c-JUN/c-JUN	1. P < 0.05 2. P < 0.01 3. P < 0.01 4. P < 0.01 5. P < 0.05 6. P < 0.01 7. P < 0.01 8. P < 0.05 9. P < 0.05 10. P < 0.05 11. P < 0.05 12. P < 0.01 13. P < 0.05
Liu et al., 2010	SD rats (male; 6/6)	230–250 g	Clamping left renal arteryand vein (ischemia; 45 min; reperfusion; 72 h)	Pentobarbital sodium (50 mg/kg)	NGR1 (40 mg/kg; i.p.) before ischemia and for 3 days after reperfusion	Receiving the same amount of saline	1. Serum levels of creatinine 2. MPO 3. Relative TNF-α band intensity 4. TUNEL-positive cells 5. Relative p38MAPK band intensity 6. NF-κB band intensity	1. P < 0.05 2. P <0.05 3. P < 0.05 4. P < 0.05 5. P < 0.05 6. P < 0.05
Li et al., 2014	SD rats (male; 6/6)	200–220 g	Clamping superior mesenteric artery (90 min/1 or 72 h)	Pentobarbital sodium (50 mg/kg)	NGR1 (10 mg/kg/h; IVgtt) for 170 min after reperfusion	Receiving the same amount of saline	1. IκB-α change (%) 2. NF-κB change (%) 3. ATP5D change (%) 4. Zonulaoccludens -1 change (%) 5. Occludin change (%) 6. Claudin-5 change (%)	1. P < 0.05 2. P < 0.05 3. P < 0.05 4. P < 0.05 5. P < 0.05 6. P < 0.05

SD rats, Sprague-Dawley rats; LAD, the left anterior descending coronary artery; SOD, superoxide dismutase; MDA, malondialdehyde; CK, creatine kinase; LDH, lactate dehydrogenase; CK-MB, creatine kinase-MB; TNF-α, tumor necrosis factor-α; GSH-Px, glutathione peroxidase; CAT, catalase; AST, aspartate aminotransferase; AAR/LV, area at risk/left ventricle;+dp/dtmax, maximum ascending rate of left ventricular pressure; −dP/dtmax, maximum descending rate of left ventricular pressure; LVSP, left ventricular systolic pressure; LVDP, left ventricular diastolic pressure; MVC, miniature blood vessel; MVD, microvascular density; VEGF, vascular endothelial growth factor; bFGF, basic fibroblast growth factor; ATP, adenosine triphosphate; AMP, adenosine monophosphate; IL-1, interleukin-1; IL-8, interleukin-8; NF-κBp65, nuclear factor-kappa Bp65; AMPK, AMP-activated protein kinase; ROCK, Rho-associated coil kinase; MYPT1, myosin phosphatase target subunit-1; NF-κBP65, nuclear factor-κBp65; IκBα, nuclear factor of kappa light polypeptide gene enhancer in B-cells inhibitor; alpha; VDUP1, vitamin D3 upregulated protein 1; GAPDH, glyceraldehyde-3-phosphate dehydrogenase; c-JNK, c-Jun N-terminal kinase; Bcl-2, B-cell lymphoma-2; MCAO; middle cerebral artery occlusion; BCCAO, bilateral common carotid artery occlusion; BDNF, brain-derived neurotrophic factor; NGR1, notoginsenoside R1; AMI, acute myocardial ischemia; bax, Bcl-2-associated X protein; RBC, red blood cell; MPO, myeloperoxidase; ICAM-1, intercellular cell adhesion molecule-1; TUNEL, TdT-mediated dUTP nick-end labeling; ADP, adenosine diphosphate; GAPDH, glyceraldehyde-3-phosphate dehydrogenase; ZO-1, Zonula occludens-1; JAM-1, recombinant junctional adhesion molecule 1; Cav-1, caveolin 1; Cav-3, caveolin 3; AAR, area at risk; T-SOD, total superoxide dismutase; IL-1β, interleukin 1 beta; CHOP, C/EBP homologous protein; GRP78, glucose regulated protein 78; PERK, protein kinase R-like ER kinase; p-PERK, phospho-protein kinase R-like ER kinase; eIf2α, eukaryotic initiation factor 2α; IRE1, inositol-requiring enzyme-1α; ATF6, activating transcription factor 6; 8-OHdG, 8-hydroxydeoxyguanosine; HO-1, heme oxygenase-1; PI3K, p-mTOR, phospho-mammalian target of rapamycin; T-mTOR, P70S6K, protein S6 kinase; P-P70S6K, phospho-protein S6 kinase; P-4EBP-1, phospho-4EBP1.

### Cell Experiments

Eleven studies involved in cell experiments between 2014 and 2018 were included, of which four studies (Wan et al., 2015; Zhou et al., 2016; Hou et al., 2017; Zhou et al., 2017) were published in Chinese and seven studies (He et al., 2014; Meng et al., 2014; Wang et al., 2016; Yu et al., 2016; Wang et al., 2017; Liu et al., 2019; Tu et al., 2018) in English. Wistar Suckling mice cardiomyocytes were used in three studies (Wan et al., 2015; Zhou et al., 2016; Zhou et al., 2017), and embryonic cardiomyoblast-derived cardiomyocytes (H9C2) of rat were used in two studies (He et al., 2014; Yu et al., 2016). The methods of establishing I/R model in cardiomyocytes include the application of hydrogen peroxide (H_2_O_2_) (Wan et al., 2015; Zhou et al., 2016; Zhou et al., 2017) and OGD/R (He et al., 2014; Yu et al., 2016). The dosages of NGR1 were 10 μmol/L in two studies (Zhou et al., 2016; Zhou et al., 2017), 20 μmol/L in one study (Yu et al., 2016), and 100 μmol/L in two studies (He et al., 2014; Wan et al., 2015). Cell viability and TUNEL-positive rate were used as outcome measures in six studies (He et al., 2014; Wan et al., 2015; Zhou et al., 2016; Yu et al., 2016; Zhou et al., 2017; Liu et al., 2019), LDH in seven studies (He et al., 2014; Meng et al., 2014; Wang et al., 2016; Zhou et al., 2016; Zhou et al., 2017; Hou et al., 2017; Tu et al., 2018), SOD in three studies (Wan et al., 2015; Zhou et al., 2016; Zhou et al., 2017), and MDA in four studies (Meng et al., 2014; Wan et al., 2015; Zhou et al., 2016; Zhou et al., 2017). The detailed characteristics of the included studies were generalized in **Table 2**.

**Table 2:** Characteristics of the 11 included cell studies.

Study (years)	Appellation (n = experimental/control group)	Organism age tissue	Primary cells or subcultured cells	Model (method)	Treatment group (method to astragal sides)	Control group	Outcome index (time)	Intergroup differences
Zhou et al., 2016	RCM (6/6)	WistarSuckling miceMyocardium	Primary cells	Received H_2_O_2_ (50 μmol/L)	NGR1 (10 μmol/L; 24 h) before molding	No treatment	1. LDH 2. SOD 3. MDA 4. Cell viability 5. Apoptosis rate 6. p-ERK1/2 7. ERK1/2 8. p-p38 9. p38	1. P < 0.01 2. P < 0.01 3. P < 0.01 4. P < 0.01 5. P < 0.01 6. P < 0.01 7. P < 0.01 8. P < 0.01 9. P < 0.01
Zhou et al., 2017	RCM (6/6)	WistarSuckling miceMyocardium	Primary cells	Received H_2_O_2_ (50 μmol/L)	NGR1 (10 μmol/L; 24 h) before molding	No treatment	1. LDH 2. SOD 3. MDA 4. Cell viability 5. Apoptosis rate	1. P < 0.01 2. P < 0.01 3. P < 0.01 4. P < 0.01 5. P < 0.01
Wan et al., 2015	RCM (6/6)	WistarSuckling miceMyocardium	Primary cells	Received H_2_O_2 _(1 mmol/L)	Received NGR1 (100 μmol/L; 24 h) before molding	No treatment	1. Cell viability 2. Apoptosis rate 3. MDA 4. SOD5. p-JNK 6. Bax 7. Bcl -2	1. P < 0.05 2. P < 0.05 3. P < 0.05 4. P < 0.05 5. P < 0.05 6. P < 0.05 7. P < 0.05
Yu et al., 2016	H9C2 (6/6)	Rat embryonic cardiomyoblast- derived H9c2 cardiomyocytes	Subcultured cells	H/R (6 h/12 h)	NGR1 (20 μmol/L; 24 h) before molding	No treatment	1. Cell viability2. Extracellular LDH3. ROS4. Relative intensity of red/green fluorescence5. PIP positive cell rate6. TUNEL-positive7. GRP78/β-actin8. P-PERK/PERK9. P- eIf2α/eIf2α10. IRE1/β-actin11. ATF6/β-actin	1. P < 0.001 2. P < 0.001 3. P < 0.01 4. P < 0.001 5. P < 0.01 6. P < 0.001 7. P < 0.01 8. P < 0.001 9. P < 0.001 10. P < 0.01 11. P < 0.001
He et al., 2014	H9C2 (6/6)	A rat cardiac myoblast cell line	Subcultured cells	OGD/R(15 h)	NGR1 (100 μmol/L)	No treatment	1. TUNEL-positive2. Cell viability3. LDH4. Bcl-2/Bax5. Cleaved caspase-3/procaspase-36. ATP7. AMP8. ATP synthase activity9. P-AMPK/β-actin10. ATPsynthase-α/β-actin11. ATP synthase-β/β-actin12. ATP 5D/β-actin13. ROCK/β-actin14. P-MYPT1/MYPT1	1. P < 0.05 2. P < 0.05 3. P < 0.05 4. P < 0.05 5. P < 0.05 6. P < 0.05 7. P < 0.05 8. P < 0.05 9. P < 0.05 10. P > 0.05 11. P > 0.05 12. P < 0.05 13. P < 0.05 14. P < 0.05
Liu et al., 2019	RCM	NeonatalSD rats Myocardium	Primary cells	OGD (6 h)	NGR1 (20 μmol/L) for 24 h	No treatment	1. Cell viability2. Apoptotic cells3. RNA level expression of miR-214. mRNA and protein levels of PTEN	1. P < 0.05 2. P < 0.05 3. P < 0.05 P < 0.05
Meng et al., 2014	Primary cortical neurons (6/6)	SD rats embryo cerebral cortices	Primary cells	OGD/R (2 h/24 h)	NGR1 (25 μM) for 24 h before ischemia	Treated with DMSO (final concentration was 0.1%)	1. Intracellular ROS2. NADPH oxidase activity3. Superoxide levels4. Mitochondrial superoxide5. MDA6. Protein carbonyl7. 8-OHdG8. TUNEL-positive cells rate9. Apoptosis rate10. Ratio of red to green fluorescence intensity11.Cell viability12. LDH13. Caspase-3 activity	1. P < 0.01 2. P < 0.01 3. P < 0.01 4. P < 0.01 5. P < 0.01 6. P < 0.01 7. P < 0.01 8. P < 0.01 9. P < 0.01 10. P < 0.01 11. P < 0.01 12. P < 0.01 13. P < 0.01
Wang, 2016	Primary cortical neurons (5/5)	SD rats embryo cerebral cortices	Primary cells	OGD/R (1.5 h/24 h)	NGR1 (10 μmol/L)	DMSO (1%)	1. Cell viability2. LDH3. Ratio of GRP78/β-actin4. Ratio of P-PERK/PERK5. Cleaved-caspase-12/caspase-126. Ratio of P-IRE1α/IRE1α7. Ratio of BCL-2/β-actin	1. P < 0.05 2. P < 0.05 3. P < 0.05 4. P < 0.05 5. P < 0.05 6. P < 0.05 7. P < 0.05
Hou et al., 2017	Primary cortical neurons (5/5)	SD rats embryo cerebral cortices	Primary cells	OGD/R (1.5 h/24 h)	NGR1 (10 μmol/L)		1. Cell viability2. LDH3. ATF6/Akt4. P-Akt/Akt5. Cleaved Caspase-3/β-actin6. Bax/β-actin	1. P < 0.05 2. P < 0.05 3. P < 0.05 4. P < 0.05 5. P < 0.05 6. P < 0.05
Wang et al., 2017	Primary cortical neuron (5/5)	SD rats embryo cerebral cortices	Primary cells	OGD/R (1.5 h/24 h)	NGR1 (10 μmol/L)	DMSO (1%)	1. Cell viability2. Ratio of p-PLCβ/PLCβ3. Ratio of p-PLCγ/PLCγ4. Ratio of IP3R1/β-actin5. Ratio of p-PERK/β-actin6. Ratio of p-IRE1/β-actin7. Ratio of CHOP/β-actin8. Ratio of p-CaMKII/β-actin9. Ratio of p-P38/β-actin10. Ratio of p-JNK/β-actin11. Ratio of TUNEL-positive cells	1. P < 0.05 2. P < 0.05 3. P < 0.05 4. P < 0.05 5. P < 0.05 6. P < 0.05 7. P < 0.05 8. P < 0.05 9. P < 0.05 10. P < 0.05 11. P < 0.05
Tu et al., 2018	Primary cortical neuron (5/5)	Rats embryo cerebral cortices	Primary cells	OGD/R (1.5 h/24 h)	NGR1 (10 μmol/L)		1. Cell viability2. LDH3. Ratio of PI3K/β-actin4. P-Akt/T-Akt5. P-mTOR/T-mTOR6. P-P70S6K/P70S6K7. P-4EBP-1/4EBP-18. P-JNK/T-JNK9. P-c-JUN/c-JUN	1. P < 0.05 2. P < 0.05 3. P < 0.05 4. P < 0.05 5. P < 0.05 6. P < 0.05 7. P < 0.05 8. P < 0.05 9. P < 0.05

RCM, rat cardiac myocytes; OGD/R, oxygen glucose deprivation/reoxygenation; LDH, lactic dehydrogenase; SOD, superoxide dismutase; MDA, TUNEL, terminal deoxynucleotidyl transferase-mediated dUTP nick end labelling; p-ERK1/2, phospho extracellular regulated protein kinases; p-JNK, phospho c-Jun N-terminal kinase; ERK1/2, extracellular regulated protein kinase; Bax, Bcl-2 Associated X protein; Bcl-2, B-cell lymphoma-2; ROS, reactive oxygen species; GRP78, glucose-regulated protein 78; P-PERK, phospho protein kinase RNA-like endoplasmic reticulum kinase; PERK, PKR-like endoplasmic reticular kinase; P-eIf2α, phospho-eukaryotic initiation factor 2α; eIf2α, eukaryotic initiation factor 2; IRE1, inositol-requiring enzyme-1α; ATF6, activating transcription factor 6; P-AMPK, phosphorylation of AMP-activated protein kinase; ROCK, Rho-associated kinase protein; P-MYPT1, phosphorylating myosin light chain phosphatase; MYPT1, myosin light chain phosphatase; ATP, adenosine triphosphate; AMP, adenosine monophosphate; SD rats, Sprague-Dawley; DMSO, dimethyl sulfoxide; OGD, oxygen-glucose deprivation; NADPH, nicotinamide adenine dinucleotide phosphate; 8-OHdG, 8-hydroxy-2’-deoxyguanosine; P-IRE1α, phosphoinositol requiring enzyme 1α; Akt, protein kinase B; P-Akt, phosphorylated Akt; p-PLCβ, phospho phospholipase Cβ; PLCβ, phospholipase C β; p-PLCγ, phospho phospholipase Cγ; PLCγ, phospholipase Cγ; IP3R1, inositol 1;4;5-triphosphate receptor type 1; p-IRE1, inositol-requiring enzyme 1; CHOP, C/EBP homologous protein; p-CaMKII, phospho Ca2+/calmodulin-dependent protein kinase II; PI3K, phosphoinositide 3-kinase; P-mTOR, phosphorylated mammalian target of rapamycin; T-mTOR, mammalian target of rapamycin; P-P70S6K, P70S6K P-4EBP-1, phosphorylated 4E-binding protein 1; 4EBP-1, 4E-binding protein 1.

In addition, seven studies (Liu et al., 2010; Meng et al., 2014; Yu et al., 2014; Xia et al., 2015; Yu et al., 2016; Zou et al., 2017; Tu et al., 2018) reported chemical analysis of NGR1 in animal studies. Seven studies (Meng et al., 2014; Wan et al., 2015; Wang et al., 2016; Wang et al., 2016; Liu et al., 2019; Tu et al., 2018) reported chemical analysis of NGR1 in cell studies. The characteristics of NGR1 were shown in **Table 3**.

**Table 3:** Statement of the characteristics of NGR1.

Study	Source	Species, concentration	Quality control reported? (Y/N)	Chemical analysis reported? (Y/N)
Liu et al., 2010	Chinese National Institute for the Control of Pharmaceutical and Biological Products	*Panax notoginseng*40 mg/kg	N	Y-HPLC
Deng and Lai 2013	Guangxi Wuzhou Pharmaceutical (Group) Co., Ltd	*P. notoginseng*, 10 mg/kg	Y(120502)	N
Han, 2014	Fengshanjian Medicine Research Co. Ltd. (Kunming, Yunnan, China)	*P. notoginseng*, 5 mg/kg	N	N
He et al., 2014	Feng-Shan-Jian Medical, Kunming, China	*P. notoginseng*, 20 mg/kg	N	N
He et al., 2014	Feng-Shan-Jian Medical, Kunming, China	*P. notoginseng*, 0.1 mM	N	N
Li et al., 2014	Feng-Shan-Jian Medical (Kunming, China)	*P. notoginseng*, 10 mg/kg	N	N
Meng et al., 2014	Shanghai Winherb Medical S & T Development (China)	*P. notoginseng*, 20 mg/kg	N	Y-HPLC
Meng et al., 2014	Shanghai Winherb Medical S & T Development (China)	*P. notoginseng*, 25 μM	N	Y-HPLC
Yu et al., 2014	Chengdu Must Bio-Technology Co., Ltd	*P. notoginseng*, 2.5 mg/kg/d	Y(MUST-23091001)	Y-HPLC
Dong et al., 2015	Nanjing ZeLang Medicine Photochemistry Technology Co., Ltd	*P. notoginseng*, 7 mg/kg	N	N
Xia et al., 2015	National Institutes for Food andDrug Control (Beijing, China)	*P. notoginseng*, 60 mg/kg	N	Y-HPLC
Wan et al., 2015	Chengdu Must Bio-Technology Co., Ltd	*P. notoginseng*, 100 μM	N	Y-HPLC
Wang et al., 2016	SigmaAldrich	*P. notoginseng*	N	N
Yu et al., 2016	Shanghai Winherb Medical S&T Development (Shanghai, China)	*P. notoginseng*, 15 mg/kg	N	Y-HPLC
Yu et al., 2016	Shanghai Winherb Medical S&T Development (Shanghai, China)	*P. notoginseng*, 20 μM	N	Y-HPLC
Zhou et al., 2016	Guangzhou Institute for drug control	*P. notoginseng*, 10 μmol/L	N	N
Wang et al., 2016	SigmaAldrich	*P. notoginseng*, 20 mmol/L	N	Y-HPLC
Hou et al., 2017	Nanjing Jiancheng Bioengineering Institute	*P. notoginseng*, 20 μmol/L	N	N
Wang et al., 2017	Sigma-Aldrich	*P. notoginseng*, 10 μmol/L	N	Y-HPLC
Zhao et al., 2017	Shanghai Yuanye Biological Technology Co. Ltd	*P. notoginseng*, 5mg/ml	N	N
Zhou et al., 2017	Guangzhou Institute for drug control	*P. notoginseng*, 10 μM	N	N
Zou et al., 2017	Shanghai Ronghe Pharmaceutical Technology Development Co., Ltd	*P. notoginseng*, 100 mg/kg	N	Y-HPLC
Liu et al., 2019	Sigma-Aldrich	*P. notoginseng*, 80μM	N	Y-HPLC
Tu et al., 2018	Sigma-Aldrich	*P. notoginseng*, 15 mg/kg	N	Y-HPLC
Tu et al., 2018	Sigma-Aldrich	*P. notoginseng*, 10 µmol/l	N	Y-HPLC

### Study Quality

#### Animal Studies

The scores of study quality ranged from 3 to 8 in a total of 10 points. All included studies were peer-reviewed publication. All the studies reported that the animals were allocated randomly to treatment or control group, and the anesthetics used in the experiments with no intrinsic organ-protective activity. However, sample size calculation, blinded induction of model, and blinded assessment of outcome were not reported in all included studies. Seven studies (He et al., 2014; Meng et al., 2014; Xia et al., 2015; Wang et al., 2016; Yu et al., 2016; Zou et al., 2017; Tu et al., 2018) stated compliance with animal welfare regulations and eight studies (He et al., 2014; Meng et al., 2014; Yu et al., 2014; Dong et al., 2015; Xia et al., 2015; Wang et al., 2016; Yu et al., 2016; Tu et al., 2018) declared no potential conflict of interests. The methodological quality of included studies was shown in **Table 4**.

**Table 4:** Risk of bias of the included *in vivo* studies.

Study	A	B	C	D	E	F	G	H	I	J	Total
Deng and Lai 2013	**√**		**√**			**√**					**3**
Han, 2014	**√**	**√**	**√**			**√**					**4**
He et al., 2014	**√**	**√**	**√**	**√**		**√**			**√**	**√**	**7**
Yu et al., 2014	**√**		**√**	**√**		**√**				**√**	**5**
Xia et al., 2015	**√**	**√**	**√**	**√**		**√**			**√**	**√**	**7**
Yu et al., 2016	**√**	**√**	**√**	**√**	**√**	**√**			**√**	**√**	**8**
Tu et al., 2018	**√**	**√**				**√**			**√**	**√**	**5**
Meng et al., 2014	**√**	**√**	**√**		**√**	**√**			**√**	**√**	7
Zou et al., 2017	**√**	**√**	**√**			**√**			**√**		**5**
Wang et al., 2016	**√**	**√**				**√**			**√**	**√**	**5**
Zhao et al., 2017	**√**	**√**				**√**					**3**
Dong et al., 2015	**√**	**√**				**√**				**√**	**4**

*Studies fulfilling the criteria of: A, peer reviewed publication; B, control of temperature; C, random allocation to treatment or control; D, blinded induction of model; E, blinded assessment of outcome; F, use of anesthetic without significant intrinsic vascular protection activity; G, appropriate animal model (aged, diabetic, or hypertensive); H, sample size calculation; I, compliance with animal welfare regulations; J, statement of potential conflict of interests.*

### Cell Studies

The scores of study quality ranged from 3 to 5 in a total of 10 points. All included studies were peer-reviewed publication. All the studies reported control of experimental conditions, the effect or safety of treatment, and statement of no potential conflict of interests. Nine studies (Meng et al., 2014; Wan et al., 2015; Wang et al., 2016; Zhou et al., 2016; Hou et al., 2017; Wang et al., 2017; Zhou et al., 2017; Liu et al., 2019; Tu et al., 2018) used primary cultured cells and two studies (He et al., 2014; Yu et al., 2016) used subcultured cells. However, sample size calculation and blinded assessment of outcome were not reported in all included studies. The methodological quality was shown in **Table 5**.

**Table 5:** Risk of bias of the included *in vitro* studies.

Study	A	B	C	D	E	F	G	H	I	J	Total
He et al., 2014	**√**			**√**	**√**					**√**	**4**
Wan et al., 2015	**√**				**√**					**√**	**3**
Zhou et al., 2016	**√**	**√**			**√**					**√**	**4**
Zhou et al., 2017	**√**	**√**			**√**					**√**	**4**
Liu, 2018	**√**	**√**		**√**	**√**					**√**	**5**
Meng et al., 2014	**√**	**√**		**√**	**√**					**√**	**5**
Wang, 2016	**√**	**√**		**√**	**√**					**√**	**5**
Wang et al., 2017	**√**	**√**		**√**	**√**					**√**	**5**
Hou et al., 2017	**√**	**√**		**√**	**√**					**√**	**5**
Tu et al., 2018	**√**	**√**		**√**	**√**					**√**	**5**

Studies fulfilling the criteria of: A, peer-reviewed publication; B, use of appropriate primary cells to study; C, cell lines with reliable source or validated by appropriate methods; ,D, assess toxicity of treatment on cells; E, culture environment (culture media/sera, pH/CO_2_ and temperature); F, random allocation to treatment or control; G, blinded induction of model; H, blinded assessment of outcome; I, calculation of the sample size necessary to achieve sufficient power; and J, statement of potential conflict of interests. Each item was awarded one point.

### Effectiveness

#### Myocardial I/R Injury

##### MI Size

**Figure 3:**
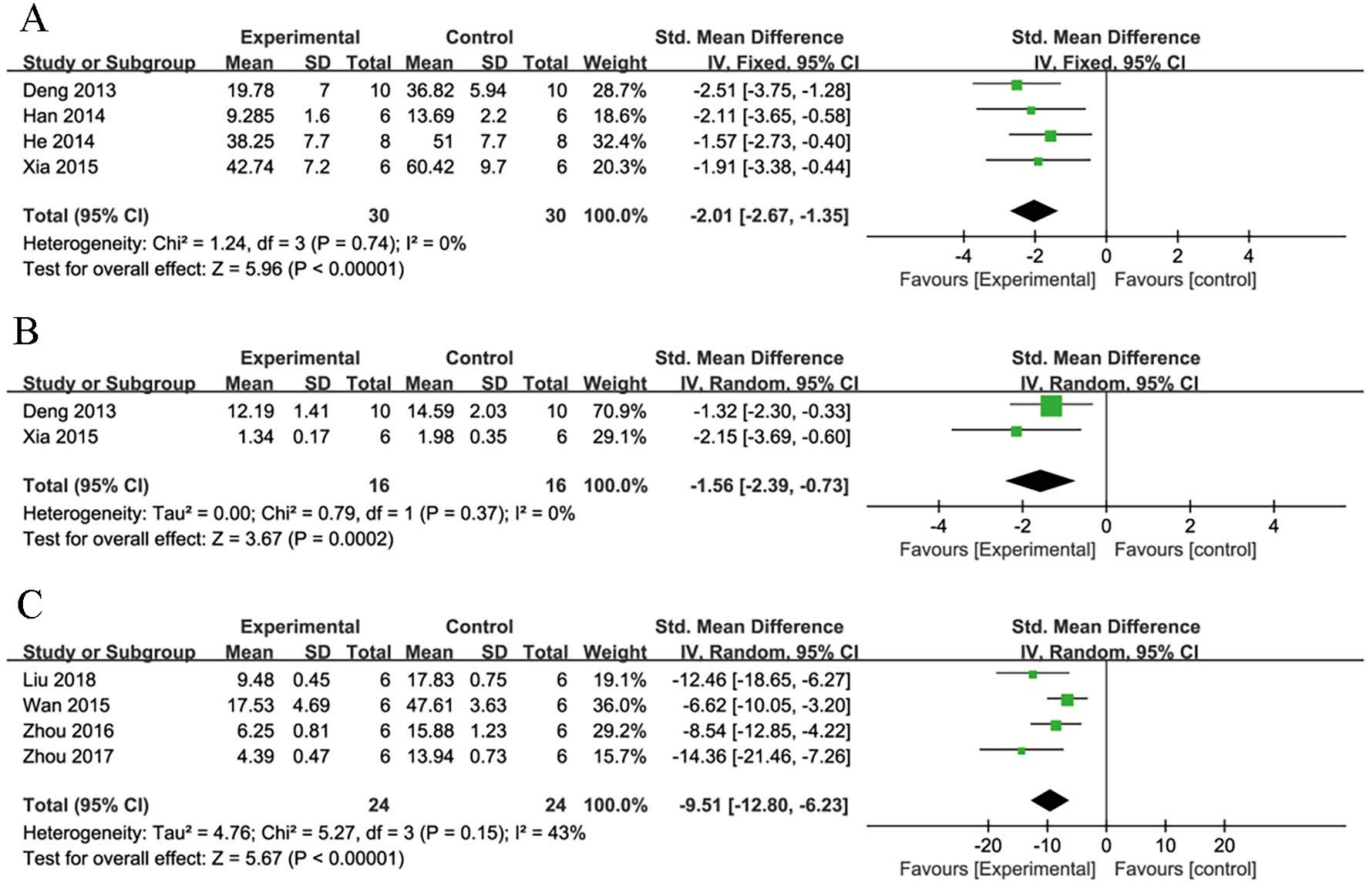
The forest plot: **(A)** effects of notoginsenoside R1 for reducing the myocardial infarction size compared with the control group (n = 30 per group). **(B)** Effects of notoginsenoside R1 for reducing the creatine kinase compared with the control group (n = 16 per group). **(C)** The forest plot: effects of notoginsenoside R1 for reducing cardiomyocytes apoptosis rate compared with the control group (n = 24 per group).

Meta-analysis of four studies (Deng and Lai, 2013; Han, 2014; He et al., 2014; Xia et al., 2015) showed NGR1 had significant effect on reducing MI size compared with control group [n = 60, SMD: -2.01, 95% CI: -2.67 to -1.35, P < 0.00001; heterogeneity χ^2^ = 1.24, df = 3 (P = 0.74), I^2^ = 0%] (**Figure 3A**).

##### Cardiac Enzyme

Meta-analysis of three studies (Deng and Lai, 2013; Xia et al., 2015; Yu et al., 2016) showed NGR1 had significant effect on decreasing CK compared with the control group [*n* = 52, SMD: -5.05, 95% CI: -8.83 to -1.28, *P* = 0.009; heterogeneity: χ^2^ = 22.94, df = 2 (*P* < 0.0001), I^2^ = 91%]. Owing to the obvious heterogeneity, we conducted a sensitivity analyses and removed one study (Yu et al., 2016) that utilized Langendroff-perfused rat hearts. Meta-analysis of the remaining two studies (Deng and Lai, 2013; Xia et al., 2015) showed NGR1 had significant effect on decreasing CK compared with the control group [*n* = 32, SMD: -2.06, 95% CI: -2.96 to -1.15, *P*< 0.00001; heterogeneity: χ^2^ = 0.02, df = 1 (*P* = 0.89), I^2^ = 0%] (**Figure 3B**). We failed to conduct meta-analysis of serum MDA in the two studies (Xia et al., 2015; Yu et al., 2016) because of high heterogeneity. However, both of them favored that NGR1 treatment could reduce the level of serum MDA compared with the control group (*P* < 0.05).

##### Cardiomyocyte Apoptosis Rate

Meta-analysis of six studies (He et al., 2014; Wan et al., 2015; Yu et al., 2016; Zhou et al., 2016; Zhou et al., 2017; Liu et al., 2019) showed NGR1 had significant effect on reducing TUNEL-positive cell rate compared with the control group [*n* = 72, SMD: -10.94, 95% CI: -14.77 to -7.11, *P* < 0.00001; heterogeneity: χ^2^ = 13.83, df = 5 (*P* = 0.02), I^2^ = 64%]. Owing to the obvious heterogeneity, we conducted a sensitivity analyses and removed two studies (He et al., 2014; Yu et al., 2016) that utilized subcultured cells. Meta-analysis of the remaining four studies (Wan et al., 2015; Zhou et al., 2016; Zhou et al., 2017; Liu et al., 2019) showed NGR1 had significant effect on decreasing TUNEL-positive cell rate compared with the control group [*n* = 48, SMD: -9.51,95% CI: -12.80 to -6.23, *P* < 0.00001; heterogeneity: χ^2^ = 5.27, df = 3 (*P* = 0.15), I^2^ = 43%] (**Figure 3C**).

##### Cardiomyocyte Viability

**Figure 4:**
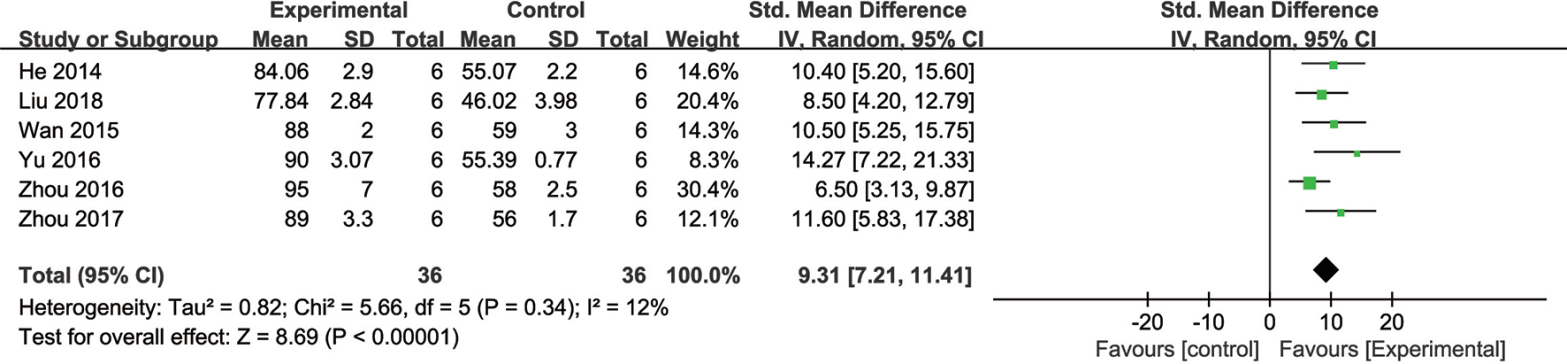
The forest plot: effects of notoginsenoside R1 for increasing cardiomyocytes cell viability compared with the control group (n = 36 per group).

Meta-analysis of six studies (He et al., 2014; Wan et al., 2015; Yu et al., 2016; Zhou et al., 2016; Zhou et al., 2017; Liu et al., 2019) showed NGR1 had significant effect on increasing cell viability compared with the control group [*n* = 36, SMD: 9.31, 95% CI: 7.21 to 11.41, *P* < 0.00001; heterogeneity: χ^2^ = 5.65, df = 5 (*P* = 0.34), I^2^ = 12%] (**Figure 4**).

##### Cardiomyocytes LDH

**Figure 5:**
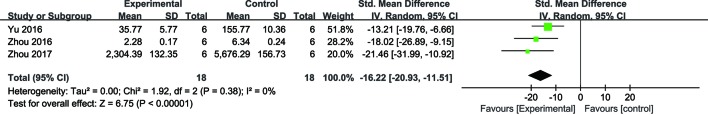
The forest plot: effects of notoginsenoside R1 for reducing cardiomyocytes LDH release compared with the control group (n = 18 per group).

Meta-analysis of four studies (He et al., 2014; Yu et al., 2016; Zhou et al., 2016; Zhou et al., 2017) showed NGR1 had significant effect on decreasing cell LDH compared with the control group [*n* = 48, SMD: -13.57, 95% CI: -21.27 to -5.88, *P* = 0.0005; heterogeneity: χ^2^ = 16.3, df = 3 (*P* = 0.001), I^2^ = 82%].Owing to high heterogeneity, we conducted a sensitivity analyses and removed one study (He et al., 2014) for non-pretreatment with NGR1. Meta-analysis of the remaining three studies (Yu et al., 2016; Zhou et al., 2016; Zhou et al., 2017) showed that NGR1 had significant effect on reducing cell LDH compared with the control group [*n* = 36, SMD: -16.22, 95% CI: -20.93 to -11.51, *P* < 0.00001; heterogeneity: χ^2^ = 1.92, df = 2 (*P* = 0.38), I^2^ = 0%] (**Figure 5**).

#### Cerebral Injury

##### Cerebral Infarction Volume

**Figure 6:**
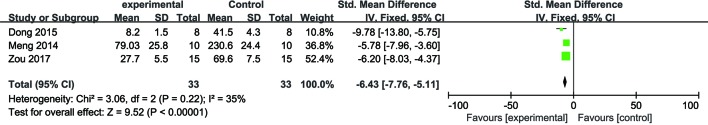
The forest plot: effects of notoginsenoside R1 for reducing cerebral infarction volume compared with the control group (n = 33 per group).

Meta-analysis of five studies (Meng et al., 2014; Dong et al., 2015; Wang et al., 2016; Zou et al., 2017; Tu et al., 2018) demonstrated NGR1 had significant effect on decreasing cerebral infarction volume compared with the control group [*n* = 94, SMD: -5.25, 95% CI: -6.24 to -4.27, *P* < 0.00001; heterogeneity: *χ* ^2^ = 13.65, df = 4 (*P* = 0.009), I^2^ = 71%]. Owing to the obvious heterogeneity, we conducted a sensitivity analyses and removed two studies (Wang et al., 2016; Tu et al., 2018) that utilized 7-day-old SD rats. Meta-analysis of the remaining three studies (Meng et al., 2014; Dong et al., 2015; Zou et al., 2017) demonstrated NGR1 had significant effect on reducing cerebral infarction volume compared with the control group [*n* = 66, SMD: -6.43, 95% CI: -7.76 to -5.11, *P* < 0.00001; heterogeneity: χ^2^ = 3.06, df = 2 (P = 0.22), I^2^ = 35%] (**Figure 6**).

##### Neurologic Deficit Score

**Figure 7:**
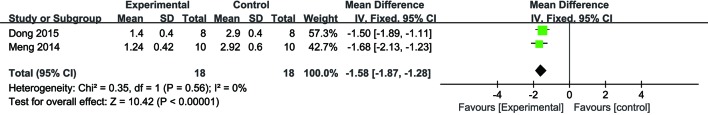
The forest plot: effects of notoginsenoside R1 for reducing neurologic deficit score compared with the control group (n = 18 per group).

Meta-analysis of two studies (Meng et al., 2014; Dong et al., 2015) demonstrated NGR1 had significant effect on reducing neurologic deficit score compared with the control group [*n* = 26, SMD: -1.58, 95% CI: -1.87 to -1.28, *P*< 0.00001; heterogeneity: χ^2^ = 0.35, df = 1 (*P* = 0.56), I^2^ = 0%] (**Figure 7**).

##### Cerebral Cell Viability

**Figure 8:**
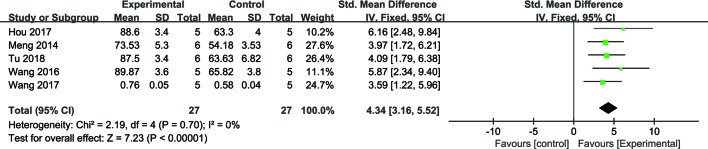
The forest plot: effects of notoginsenoside R1 for increasing cerebral cell viability compared with the control group (n = 27 per group).

Meta-analysis of five studies (Wang et al., 2016; Wang et al., 2017; Hou et al., 2017; Tu et al., 2018) demonstrated NGR1 had significant effect on increasing cell viability compared with the control group [*n* = 54, SMD: 4.34, 95% CI: 3.16 to 5.52, P < 0.00001; heterogeneity: χ^2^ = 2.19, df = 4 (P = 0.70), I^2^ = 0%] (**Figure 8**).

#### Renal Injury

One study (Liu et al., 2010) reported that NGR1 had significant effect on reducing MPO and serum creatinine compared with the control group (P < 0.05).

#### Intestinal Injury

One study (Li et al., 2014) reported that NGR1 had significant effect on reducing MPO and Park/Chiu score compared with the control group (P < 0.05).

#### Organ-Protection Mechanisms

**Figure 9:**
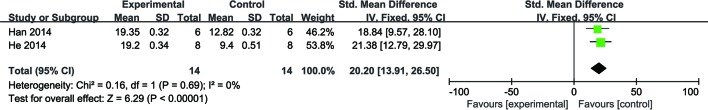
The forest plot: effects of notoginsenoside R1 for increasing ATP compared with the control group (n = 14 per group).

**Figure 10:**
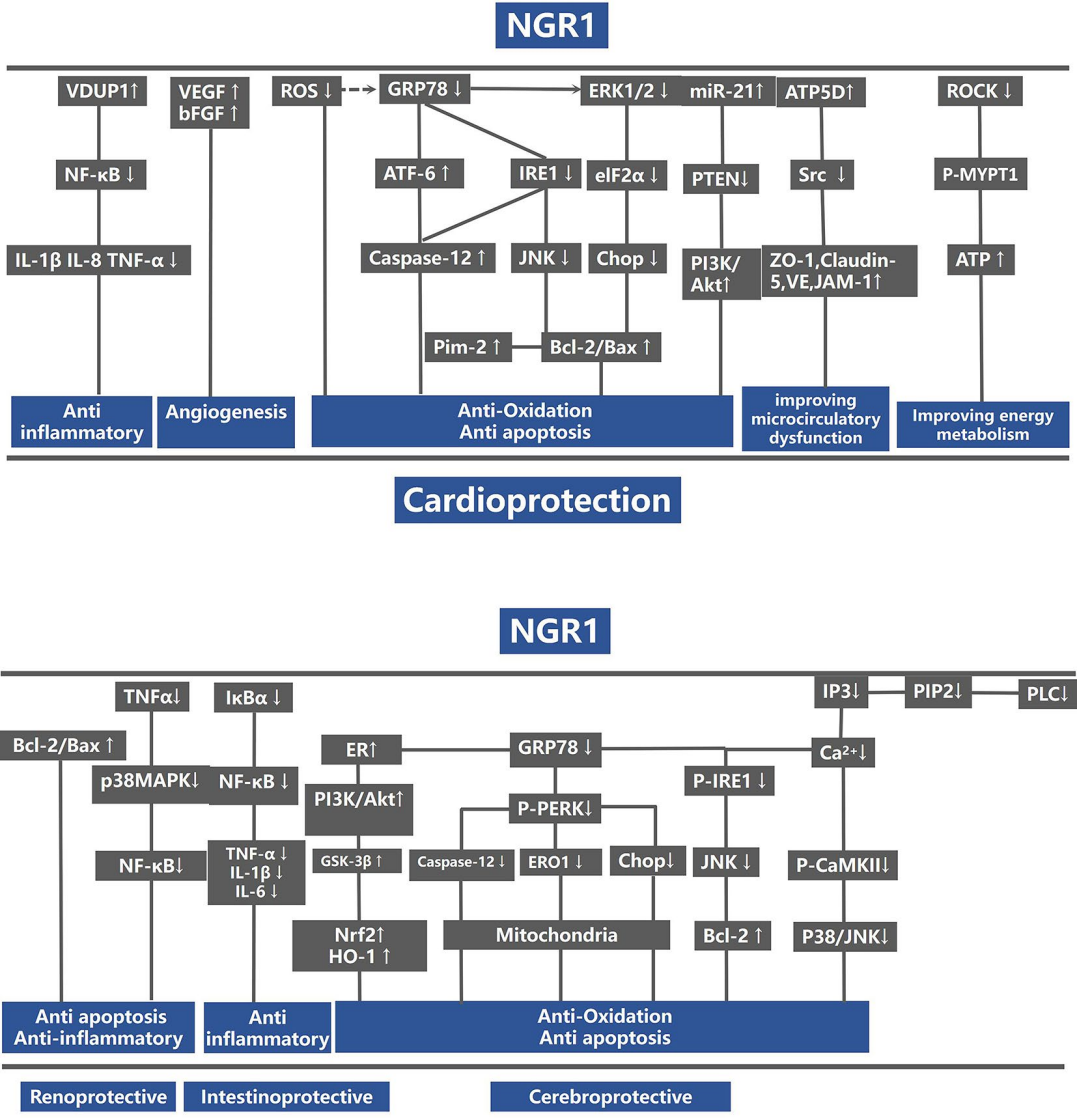
A schematic representation of cardioprotective mechanisms of NGR1 for organs ischemia/reperfusion injury. VDUP1, vitamin D3 up-regulated protein 1; NF-κB, nuclear factor-kappa; IL-1, interleukin-1; IL-1β, interleukin 1 beta; IL-8, interleukin-8; TNF-α, tumor necrosis factor-α; VEGF, vascular endothelial growth factor; bFGF, basic fibroblast growth factor; GRP78, glucose regulated protein 78; PERK, protein kinase R-like ER kinase; ATF-6, activating transcription factor 6; IRE1, inositol-requiring enzyme-1α; JNK, Jun N-terminal kinase; CHOP, C/EBP homologous protein; B-cell lymphoma-2; Bax, BCL2-associated X protein; eIf2α, eukaryotic initiation factor 2α; PTEN, phosphatase and tensin homolog deleted on chromosome ten; PI3K, p-mTOR, phospho-mammalian target of rapamycin; ZO-1, Zonula occludens-1; JAM-1, recombinant junctional adhesion molecule 1; Cav-1, caveolin 1; Cav-3, caveolin 3; MYPT1, myosin phosphatase target subunit-1; ATP, adenosine triphosphate; ROCK, Rho-associated kinase; GSK-3β, glycogen synthase kinase-3 beta; IκBα, nuclear factor of kappa light polypeptide gene enhancer in B-cells inhibitor; alpha; HO-1, heme oxygenase-1; Nrf2, nuclear factor erythroid 2-related factor 2; IP3, inositol 1, 4, 5-trisphosphate; PLC, phospholipase C.

For animal experiments, meta-analysis of two studies (Han, 2014; He et al., 2014) showed NGR1 had significant effect on increasing ATP compared with the control group [*n* = 28, SMD: 20.20, 95% CI: 13.91 to 26.50, *P* < 0.00001; heterogeneity: χ^2^ = 0.16, df = 1 (*P* = 0.69), I^2^ = 0%] (**Figure 9**). One study (He et al., 2014) showed that NGR1 increased the proportion of anti-apoptosis proteins such as Bcl-2/Bax and cleaved caspase-3/procaspase-3 compared with the control group (*P* < 0.05); one study (Yu et al., 2014) showed that NGR1 increased the density of neovasculature and improved the expression of angiogenesis protein such as VEGF and bFGF compared with the control group (*P* < 0.05); one study (He et al., 2014) showed that NGR1 improved the production of ATP and ATP5D compared with the control group (*P* < 0.05); one study (He et al., 2014) demonstrated that NGR1 reduced the protein level of phosphorylation-AMP-activated protein kinase (P-AMPK), Rho-associated coil kinase (ROCK), and phosphorylation-myosin phosphatase target subunit-1 (P-MYPT1) compared with the control group (*P* < 0.05); one study (Xia et al., 2015) indicated that NGR1 decreased the inflammatory mediators such as IL-1β and IL-8 compared with the control group (*P* < 0.05); one study (Xia et al., 2015) indicated that NGR1 decreased the expression of phosphorylation-nuclear factor-κBp65 (p-NF-κBp65) and phosphorylation-nuclear factor of kappa light polypeptide gene enhancer in B-cells inhibitor alpha (p-IκBα) compared with the control group (P < 0.05); one study (Yu et al., 2016) indicated that NGR1 lowered the level of ERS-responsive proteins, such as glucose regulated protein 78 (GRP78), phospho-protein kinase R-like ER kinase (P-PERK), activating transcription factor 6 (ATF6), and inositol-requiring enzyme-1α (IRE1) compared with the control group (*P* < 0.05); one study (Meng et al., 2014) indicated that NGR1 decreased the expression of 8-hydroxy-2 deoxyguanosine (8-OHDG) compared with the control group (*P* < 0.05); one study (Tu et al., 2018) demonstrated that NGR1 increased the synthesis of ribosomal translation regulatory proteins such as phospho-mammalian target of rapamycin (P-mTOR), phospho-protein S6 kinase (P-P70S6K), and phospho-eukaryotic initiation factor 4E binding protein 1 (P-4EBP-1) compared with the control group (*P* < 0.05); one study (Tu et al., 2018) demonstrated that NGR1 reduced the expression of pro-apoptotic proteins, such as phospho-c-Jun N-terminal kinase (P-JNK) compared with the control group (*P* < 0.05); one study (Zhou et al., 2016) showed that NGR1 decreased the level of extracellular regulated protein kinases (ERK1/2) and p38-mitogen-activated protein kinase (p38MAPK) compared with the control group (*P* < 0.01). We summarized a schematic representation for the possible intrinsic mechanisms of NGR1 protection for organ I/R injury (**Figure 10**).

## Discussion

### Summary of Evidence

To our knowledge, this is a first SR to assess the preclinical evidences of NGR1 for I/R injury both *in vivo* and *in vitro*. Twenty-five studies with 304 animals and 124 cells were selected. The quality of the included studies was generally moderate. In the present study, NGR1 exerts multiple organ protection in I/R injury, mainly through antioxidant, anti-apoptosis, and anti-inflammatory, promoting angiogenesis and improving energy metabolism.

### Limitations

None of these studies had used animals with comorbidities such as diabetes, hypertension, or hyperlipidemia. Primary cells were considered to be the suitable subjects to validate organ protective function *in vitro*, while only two studies (Xia et al., 2015; Yu et al., 2016) used subcultured cells (H9C2). None of these studies reported the blindness of ischemia induction, randomized allocation to treatment, or control group and sample size calculation. In most studies, the signal pathways investigated could not fully validate the therapeutic targets.

### Implications

Our findings demonstrated that NGR1 could attenuate I/R-induced organ injuries by a variety of pathways. For myocardial I/R injury, NGR1 could reduce MI size, increase cardiomyocyte viability, and exert cardioprotective function by the following mechanisms: 1) anti-inflammatory effect by activating the VDUP1/NF-κB signaling pathway (Xia et al., 2015) and decreasing the expression of ICAM-1 and CD18 (Han, 2014); 2) improving energy metabolism *via* up-regulation of ROCK signaling pathway (He et al., 2014); 3) promoting angiogenesis by increasing the expression of VEGF and bFGF (Yu et al., 2014); 4) inhibiting apoptosis by decreasing the expression level of ERS-responsive proteins such as GRP78, P-PERK, ATF6, and IRE, and reducing the expression of pro-apoptosis proteins such as CHOP, Caspase-12, and P-JNK (Yu et al., 2016); and 5) improving microvascular dysfunction by increasing the expression of tight junction proteins such as ZO-1, VE, JAM-1, and Claudin-5 and reducing the level of embrane intrinsic protein of Cav-1 and Cav-3 (Han, 2014). For cerebral I/R injury, NGR1 could decrease cerebral infarction volume (Meng et al., 2014; Dong et al., 2015; Wang et al., 2016; Zou et al., 2017; Tu et al., 2018) and neurological deficit score (Meng et al., 2014; Dong et al., 2015) and increase cerebral cell viability (Wang et al., 2016; Hou et al., 2017; Wang et al., 2017; Tu et al., 2018). The neuroprotective effect of NGR1 was mainly due to anti-apoptosis effect by regulating the Akt/Nrf2 and Akt/mTOR/JNK pathways (Tu et al., 2018). For renal I/R injury, NGR1 had an anti-inflammatory effect through the p38MAPK/NFκB pathway and also increased the proportion of bcl-2/bax, which could produce anti-apoptosis effect (Liu et al., 2010). For intestinal I/R injury, NGR1 could attenuate the production of inflammatory cytokine by inhibiting the NF-κB pathway and reduce the expression of loss of tight junction proteins such as zonula occluden-1 (ZO-1), occludin, and claudin-5. It was also reported that NGR1 could improve the energy metabolism *via* depressing ATP5D expression during intestinal I/R injury (Li et al., 2014). Although NGR1 could exert organ-protective effect in I/R injury *via* multiple signal pathways, the validation of NGR1 therapeutic target is insufficient. In the process of drug discovery, it is common to perform target validation to illustrate 1) the function of the potential target in disease phenotype 2) and the therapeutic efficacy of drug-like molecules through modulating the activities of the potential target (Zhu et al., 2012; Gashaw et al., 2012). The approach of validation generally included gene knockout/knockdown *in vivo*, pharmacological inhibitors, and small interfering RNA (SiRNA) of cells *in vitro* (Overall and Kleifeld 2006). In the present study, four studies (He et al., 2014; Meng et al., 2014; Yu et al., 2016; Tu et al., 2018) used signal pathway inhibitors to validate the drug targets. However, the remaining studies were not verified. Although inhibitors enjoy strong suppression functions and high selectivity, the severe side effects in organisms mean that this is not the best way to study potential targets for drugs (May et al., 2011; Yan 2016). Owing to different isoforms may hold different functions, gene knockout technique effectively eliminates all isoforms possessing alternative splicing of the precursor messenger RNAs (mRNAs) and post-translational modifications compared with RNA interference (Smith, 2003). Unfortunately, it is widespread that off-target effects highly occurred in gene knockdown (Kok et al., 2015). Thus, a call for researchers should utilize animal with gene knockout to validate the drug target.

A multitude of preclinical researches showed that many available medical treatment strategies share good efficacy on animal models, while they have poor efficacy in clinical practice (Worp and Sandercock, 2012). High-quality preclinical study can provide vital information for justifying clinical advancement (Henderson et al., 2013). An SR of preclinical research is a novel method that offers important information for future clinical trials. According to CAMARADES 10-item checklist, the score of the included studies was medium. They are similar to other animal studies, and the main limitations were that no study reported sample size calculation, allocation concealment, and blinding of outcome assessment (Bao et al., 2018). Inadequate sample size could not reach the approximately threshold with sufficient power and efficacy (Bacchetti 2010). Blinding of the ischemia model refers to establishing ischemia models for experimental animals firstly and then assigning randomly. This method can avoid selection bias in allocating the animals to the treatment groups and makes it more likely that the intergroups are comparable (Festing and Altman, 2002). An overview study involving seven meta-analyses demonstrated that unblinded induction of ischemia had a greater effect size (about 13.1%) than studies that included blinding (Crossley et al., 2008). Thus, the poor blinding of the ischemia model induction and outcome evaluation may lead to overestimating efficacy in preclinical studies (Crossley et al., 2008). Those who were attacked with IHD tend to possess an advanced age and coexist with diabetes mellitus, hyperglycemia, and hypertension (Heusch 2017; Davidson et al., 2019). However, investigators prefer to utilize young healthy animals to conduct research. This may overestimate the efficacy of intervention compared with clinical administration (Sanne et al., 2014). Accordingly, researchers should take the following factors into account: 1) improving the quality of study design; 2) blinding for induction of ischemia models and evaluation of outcomes; 3) reporting completely experimental program (sample size calculation, allocation concealment) and result; and 4) utilizing animals with comorbidities that can maximally mimic the ischemia patients suffering from hypertension, hyperlipidemia, and diabetes.

The animal model that holds maximum replication of important functional, structural, and molecular pathological characteristics of human disease is crucial to clinical translation (Saulnier-Blache et al., 2018). LAD ligation in rats is the most common I/R injury model (Nishina et al., 2001; Macarthur et al., 2013). MCA occlusion is a widely accepted model to mimic human stroke and to explore the mechanism of stroke (Durukan and Tatlisumak 2007). In the present study, most of the included studies selected these two recognized models, except one study (Deng and Lai, 2013) by injecting pituitrin and three studies (Wang et al., 2016; Tu et al., 2018; Zou et al., 2017) *via* ligating one or bilateral common carotid artery. Small animals such as mice and rats are the most commonly used vertebrate species because of their small size, low cost, easy handling, and fast reproduction rate (Seabrook et al., 2017). However, compared with humans, small animals have some limitations with small body size, short lifetime, and fundamentally distinct physiology (Cibelli et al., 2013). Large animal models are highly similar with human anatomy and pharmacodynamics, which can provide a choice of appropriate animal models for particular human disease conditions and medical applications (Van Hout et al., 2015). However, it also has weaknesses such as more expensive, difficult to manipulate, and ethical issues. Thus, we should select an ideal model that usually is a biological representative of human disease, inexpensive, reproducible, easily manipulated, and ethically sound according to experimental purpose.

A powerful outcome measure is essential to certify the efficacy of new therapy (Hietamies et al., 2018). In MI research, LVEF is the strongest predictor factor for post-MI both clinically (Barthel et al., 2013) and preclinically (Kanelidis et al., 2017). Cardiac troponins, especially the cTnI and cTnT, are the preferred biomarkers for the diagnosis and prognosis of MI clinically (Thygesen et al., 2012; Hall et al., 2015). Plasma cTnI can also serve as a predictor for myocardial injury in small animal models (Frobert et al., 2015). In cerebral ischemia, perfusion-weighted magnetic resonance imaging is an identified predictor, which can evaluate the ischemic stroke patients with perfusion abnormalities (Sasaki et al., 2011; Powers et al., 2018). In renal ischemia, GFR is an essential indicator to predict prognosis for renal injury, which can reflect the efficacy of intervention through renal blood flow and regional perfusion in the clinical setting (Dagher et al., 2003). However, few present included studies selected these important outcomes. Thus, they should be selected in priority of further myocardial I/R injury studies.

Primary cell with wild-type and unadulterated nature more closely mimics the physiological state of cells *in vivo* and generates more relevant data representing living systems (Astashkina et al., 2012; Egger et al., 2013). In nerve cell I/R models, all cells were harvested from the cerebral cortices of rat fetuses. However, in cardiomyocytes I/R models, two studies (He et al., 2014; Wan et al., 2015) used cell lines (H9C2), which did not accurately simulate the physiological and pathological state of the cells *in vivo*. Cell viability examination is a routine assay that can quantitatively assess cell reaction to drug administration. Meanwhile, the optimal drug concentration is vital for pharmacological research in cellular models, which can achieve maximum efficacy while ensuring drug safety, providing a reference for further *in vivo* experiments (Astashkina et al., 2012; Bao et al., 2018). 3-(4,5-Dimethyl-2-thiazolyl)-2,5-diphenyl-2Htetrazolium bromide (MTT) and Cell Counting Kit-8 (CCK-8) are two classical assay methods used to detect cell viability and sift the optimal drug concentration. Eight studies (Zhou et al., 2016; Wan et al., 2015; Yu et al., 2016; Wang et al., 2016; Hou et al., 2017; Wang et al., 2017; Zhou et al., 2017; Tu et al., 2018) used MTT assay, and two studies (He et al., 2014; Meng et al., 2014; Liu et al., 2019) used the CCK-8 assay. However, one study (He et al., 2014) did not conduct the assay of screening the optimal drug concentration. OGD/R is a good method for mimicking the general pathophysiological process of I/R injury at the cellular level (Jones et al., 2004). However, there are various methods for constructing cell hypoxia. Many home-made devices or chemical agents are used to create hypoxia environment. The oxygen concentration is not uniform, and the time of hypoxia varies greatly, which makes the research results to lack comparability. In addition, all included studies did not report allocation concealment, blinding of model induction, and blinding of outcome assessment. Thus, we recommend that researchers should utilize primary cells and the appropriate methods of induction in study, select optimal drug concentration, and report experimental protocols with complete information. Standardized preclinical research reporting, suitable animal/cell models, and appropriate primary outcome measures are crucial to translation from bench to bed. In order to better explore the protective function of NGR1 in I/R injury of multiple organs, researchers should add more animal/cell experiments in other less studied organs such as the liver, lung, renal, and intestinal ischemia.

To date, there has been no available therapy clinically that can alleviate I/R injury (Cung et al., 2015). An enduring ischemia leads to irreversible damage for cardiomyocytes and neurons with the low ability to regenerate or renew (Galluzzi et al., 2009; Jennings 2013). And delayed reperfusion is associated with high mortality in patients after primary PCI (Terkelsen et al., 2010). In the present study, seven studies (Deng and Lai, 2013; Li et al., 2014; Li et al., 2014; Han, 2014; He et al., 2014; Meng et al., 2014; Dong et al., 2015; Xia et al., 2015) had reported that experimental subjects were pretreated with NGR1 before modeling, and the rest of the studies had not exploited pretreatment methods. Owing to the slow onset time of herbal efficacy, administration of herbs before the induction of models was often used for herbal pharmacological researches in order to reach the effective plasma concentration. Thus, pretreatment of NGR1 before molding establishment exerts a therapeutical role rather than an approach of preventive treatment. In addition, the treatment at post-model induction is more in line with clinical practice. Definitely, it is more appropriate to study herbal pharmacology in both two different administrations.

NGR1 belongs to 20(S)-protopanaxatriol type ginsenoside, which possesses glycosides moieties at C-6 and/or C-20. NGR1 has a poor bioavailability due to fast elimination from plasma and low permeability (Zhang et al., 2019). And the direct absorption of natural ginsenosides is quite not easy due to the fact that they need to be transformed into secondary saponins *via* the metabolism of gut, and then they can be easily absorbed and utilized in the blood (Liu et al., 2009). Previous study showed a low permeability of the main metabolites of NGR1 across the Caco-2 cell monolayer, which implies a poor absorption (<1%) in humans after being taken orally; however, the permeability of the metabolites of NGR1 in intestinal bacteria is higher than that in the Caco-2 cell monolayer (Ruan et al., 2010). These findings may suggest that metabolites of NGR1 forming by intestinal bacteria have a better effect than NGR1. They are eliminated from the gastrointestinal tract to produce a series of disaccharide glycosides, including ginsenoside Rg1, notoginseng saponins R2, ginseng saponins Rh1, ginsenosides F1, and glycoside ligand proglycerol (Liu et al., 2009). The four metabolites of NGR1, including ginsenoside Rg1, notoginsenoside R2, ginsenoside Rh1, ginsenoside F1, and the aglycone protopanaxatriol, have been identified and display relative high exploration in rat plasma (Zhang et al., 2019). *In vitro* experiments mainly include the following functions: 1) carrying on experiment in Caco-2 cell to evaluate the absorption of compounds through the inner layer of the gastrointestinal tract (Artursson et al., 2001); 2) identifying the disposition of compounds among organs to study the distribution mechanism; and 3) studying and quantitating the metabolism of chemicals (Pelkonen and Turpeinen 2007). The role of experimental studies *in vitro* of the review mainly sifts the optimum concentration and explores the signaling pathway, while experimental studies *in vivo* mainly focus on the change of infarct size and the indicator relating to clinical trials (such as creatine kinase, neurologic deficit score). Previous studies have shown that the results of intervention in vitro extrapolating to the observation in vivo remains very challenging. (Wienkers and Heath, 2005; Joris et al., 2013; Hadjidemetriou et al., 2015). In the present review, the included studies did not test the metabolites of NGR1 to explore the protection mechanism on ischemic diseases. Recent studies have shown that ginsenoside metabolites share better biological effects than those of ginsenosides (Feng et al., 2017). Studies have shown that the metabolites of ginsenosides Rb1 can form rare ginsenosides such as Rg3, Rd, F2, and compound K with high bioactive functions by physical and biological treatments (Oh and Kim 2016; Fu et al., 2017). Ginsenosides Rg3 has a strong protection on cerebral ischemia compared with other ginsenosides such as Rg1, Rh2, and Rg5 (Cheng et al., 2019). Compound K showed great protection on Alzheimer’s disease and cerebral ischemia *via* enhancing cognition effects and decreasing inflammatory biomarkers (Oh and Kim 2016), and has protective effects on myocardial IR injury *via* reducing infarct size and activating the PI3K pathway (Tsutsumi et al., 2011). Similarly, ginsenoside and notoginsenoside belong to same group of triterpenoid saponins, and thus we extrapolated that NGR1 should own the same property: the metabolites of NGR1 have a greater efficacy than NGR1. Owing to the lack of direct evidence of the metabolites of NGR1, they should be further tested directly in order to extend the findings of animal models to the clinic. Currently, the application of metabolomics in herbal medicine is a research focus (Wang et al., 2017; Bi et al., 2017). So, further study should test the metabolites of NGR1 directly and one of the proper methods through using metabolomics.

## Conclusion

The findings of the present study demonstrated that NGR1 exerts organ protective functions for I/R injury, mainly through antioxidant, anti-inflammatory, and anti-apoptosis, increasing energy metabolism and angiogenesis. Further translation studies are needed.

## Data Availability Statement

The datasets generated for this study are available on request to the corresponding author.

## Author Contributions

QT, P-CZ, ZZ, L-HD, Z-HW, HZ, G-QZ, and YW designed the study. ZZ, L-HD, and Z-HW collected the data. QT, P-CZ, HZ, and ZZ performed all analyses. QT and P-CZ wrote the manuscript.

## Funding

This work was supported by the grant of the National Natural Science Foundation of China (81573750/81473491/81173395/H2902).

## Conflict of Interest

The authors declare that the research was conducted in the absence of any commercial or financial relationships that could be construed as a potential conflict of interest.
